# Supplementary material for: The impact of “male clinics” on health-seeking behaviors of adult men in rural Kenya
Source: PLoS One. 2019 Nov 21;14(11):e0224749. doi: 10.1371/journal.pone.0224749 (PMC6872147; doi:10.1371/journal.pone.0224749)
Supplement: S1 Appendix — This is the form that was filled out by Male Clinics staff at each patient visit. (DOCX) [file pone.0224749.s001.docx]

| **DATE (dd/mm/yy)** | **PATIENT #** | **AGE** | **HIV STATUS KNOWN (Y/N)**  **[If NO, offer PITC]** | **REASON FOR VISIT** | **SERVICES OFFERED** | **TESTS PERFORMED** | **TREATMENT PROVIDED** | **1^ST^ VISIT TO MALE CLINIC (Y/N)** | **STAFF INITIALS** |
| --- | --- | --- | --- | --- | --- | --- | --- | --- | --- |
|  |  |  |  |  |  |  |  |  |  |
|  |  |  |  |  |  |  |  |  |  |
|  |  |  |  |  |  |  |  |  |  |
|  |  |  |  |  |  |  |  |  |  |
|  |  |  |  |  |  |  |  |  |  |
|  |  |  |  |  |  |  |  |  |  |
|  |  |  |  |  |  |  |  |  |  |
|  |  |  |  |  |  |  |  |  |  |
