# Supplementary material for: The impact of “male clinics” on health-seeking behaviors of adult men in rural Kenya
Source: PLoS One. 2019 Nov 21;14(11):e0224749. doi: 10.1371/journal.pone.0224749 (PMC6872147; doi:10.1371/journal.pone.0224749)
Supplement: S2 Appendix — This is the interview guide used for focus group discussions. (PDF) [file pone.0224749.s002.pdf]

Recruitment Script AAAM0256

“We would like to find out why men in the Sauri community do not access health services, so we can hopefully solve the problems and offer better services for men. May I tell you about a public health study we are participating in as part of the Millennium Villages Project?”

If the participant answers yes, the staff member will read the consent form with the participant and discuss any questions and concerns that the participant may have.
