# Supplementary material for: The impact of “male clinics” on health-seeking behaviors of adult men in rural Kenya
Source: PLoS One. 2019 Nov 21;14(11):e0224749. doi: 10.1371/journal.pone.0224749 (PMC6872147; doi:10.1371/journal.pone.0224749)
Supplement: S3 Appendix — This is the questionnaire used during focus group discussions. (PDF) [file pone.0224749.s003.pdf]

IRB-AAAM0256 QUESTIONNAIRE

Questionnaire for Men in Sauri Cluster

1. Have you been to a health center before for your own health?
    a. Yes:
        i. How many times in the last year?
        ii. How many times in the last five years?
        iii. How recently?
        iv. Do you remember where you went? Check all that applies
            1. Health post?
            2. Clinic?
            3. Hospital?
            4. District Hospital?
        v. What did you go for during the last visit? Check all that applies
            1. Fever
            2. Cough
            3. Diarrhea/abdominal pain
            4. Headache
            5. Trauma/bleeding
            6. Other
        vi. How long did you wait before you decided to go to the clinic?
        vii. Do you usually go to the clinic when you are sick?
        viii. Do you go to the clinic when you are well but want to prevent illness?
    b. No:
        i. Why not?
        ii. Can you rank the highest barrier to your access to the health center amongst:
            1. Distance
            2. Cost of services
            3. Cost of transport
            4. No time
            5. Don't think it will be helpful to go to the health center
        iii. When you are sick, where do you go? What do you do?
        iv. What would make you come to a clinic?
        v. Do members of your family, such as spouse or children, use the clinics?

2. Are you satisfied with the providers who work at the clinic?
    a. With their attitude towards clients?
    b. With their competency?
    c. Are there improvements that could be made to the staff?
3. Who in your community most frequently comes to the clinic? Children? Women? Men? Older community members? Others?
4. If a woman or child needs to go to the clinic, do you think it is important for the husband or father to go to the clinic with them? Why or why not?
5. What do you believe are the most common reasons that people use the clinics?
    a. What about men in particular?
6. In your opinion, what (if any) are the best services offered by the clinic?
7. In your opinion, which (if any) of the services offered at the clinic could be improved? How?
8. Can you give an example of services that you would like to see offered at the clinic that aren't currently offered?
9. What would make you more likely to come to the clinic?
    a. When you are sick and need services for your own health
    b. When you are well but want to prevent illness
    c. When you are well and want to access other services (Family Planning…)
10. Has a Community Health Worker ever spoken with you, either as an individual or in a group, about services at the clinic?
    a. Yes:
        i. Did that make you want to come to the clinic?
    b. No:
        i. Would CHWs talking to you make you more likely to come to the clinic?
11. Do you have any fears about coming to the clinic?
12. Do you have any questions for us?
