# Supplementary material for: The impact of “male clinics” on health-seeking behaviors of adult men in rural Kenya
Source: PLoS One. 2019 Nov 21;14(11):e0224749. doi: 10.1371/journal.pone.0224749 (PMC6872147; doi:10.1371/journal.pone.0224749)
Supplement: S4 Appendix — This is the consent form used to recruit patients for the study. (PDF) [file pone.0224749.s004.pdf]

IRB-AAAM0256
Consent Form

COLUMBIA UNIVERSITY
INFORMED CONSENT DOCUMENT

Assessing Adult Males' Views Towards Health Clinics – Informed Consent for Adult Males

Research Supervisor: Yanis Ben Amor (The Earth Institute, Columbia University);

Student Researcher: Eric McFeely (Columbia University College of Physicians and Surgeons)

Department: The Earth Institute, Millennium Villages Project, Sauri Kenya

Telephone: +254 708090234

INVESTIGATOR'S STATEMENT

You are being asked to participate in this survey sponsored by the Millennium Villages Project (MVP). The purpose of this consent form is to give you the information you will need to help you decide whether or not to be in the survey. This survey will be read to you for you to answer. You may ask questions about the purpose of the research, what we would ask you to do, the possible risks and benefits to you and to your community, your rights as a volunteer, and anything else about the research or this form that is not clear. When all your questions have been answered, you can decide if you want to be in the survey or not. This process is called 'informed consent'.

PURPOSE

Millennium Villages Project (MVP) has identified that there is an underutilization in the community of the health clinics in Sauri by men like yourself. This situation likely persists because MVP is not familiar with the attitudes of men in the community towards the health centers and how the health centers could best serve the needs of men in your community. The purpose of this survey is to formally assess men's opinions on why they do or do not visit the health centers and how the health centers in Sauri could provide services that would better serve men. It is our hope that by identifying what obstacles exist in the men's attitudes towards health centers we can better develop programs that will encourage the use of the health centers by the men in the community.

BENEFITS

The information obtained from this survey might be beneficial to you, to your children and to your community.

PROCEDURES

You have been chosen to participate in this survey because you are an adult man who may have opinions about the health centers in Sauri. Your participation in the survey will be limited to the questions you answer during the survey.

All interviewing will be done by a member of the MVP staff. After all the men participating in the study have been surveyed, the Student Researcher and the research team from Columbia University will compile their answers to develop a consensus about attitudes towards the health centers. You are not obligated to complete the survey and may discontinue participation at any time.

RISKS, STRESS, OR DISCOMFORT

There is an element of discomfort associated with answering questions about your private views. Should you feel that this survey causes you any undue discomfort, you may discontinue participation.

OTHER INFORMATION

All information gathered for the survey will be will be anonymous.

PARTICIPATION AND CONFIDENTIALITY

Your participation is voluntary. You may refuse to participate or withdraw from participation at any time during the survey. We will not ask you to give any personal information for this survey. Your answers are completely anonymous.

PRIVATE INFORMATION

Private information will not be included in this survey. Your results will be completely anonymous.

CONTACT INFORMATION

If at any time you have questions regarding the research or your participation, you should contact the local investigator for Kenya, Eric McFeely, who will answer all questions. Mr. Eric McFeely's telephone number is +254 (to be updated on the ground). You should also contact a member of the research staff if you have any concerns or complaints about the research.

If at any time you have comments regarding the conduct of this research or questions about your rights as a research participant, you should contact the Institutional Review Board (IRB) Administrator for Columbia University at +1-212-851-7040.

PARTICIPANT'S STATEMENT

I have read the above purpose of the survey, and understand my role in participating in the research. I volunteer to take part in this research. I have had a chance to ask questions. If I have questions later about the research, I can ask the local investigator listed above. I understand that I may refuse to participate or withdraw from participation at any time without consequence to myself. The principal and local investigator may withdraw me at his/her professional discretion. If I have questions about my rights as a research participant, I can call the Institutional Review Board office for Columbia University at +1 (212) 851-7040. I certify that I am 18 years of age or older and freely give my consent to participate in this survey. I will receive a copy of this document for my records.

Circle Yes if you agree to participate.
Circle No if you do not agree to participate.

YES NO

INVESTIGATOR'S STATEMENT

I have discussed the proposed research with this participant, and in my opinion, the participant understands the benefits, risks and alternatives (including non-participation) and is capable of freely consenting to participate in the research.

Signature ________________________________ Date: _________________
Member of the Research Team

Print Name: ______________________
