# Supplementary material for: The impact of “male clinics” on health-seeking behaviors of adult men in rural Kenya
Source: PLoS One. 2019 Nov 21;14(11):e0224749. doi: 10.1371/journal.pone.0224749 (PMC6872147; doi:10.1371/journal.pone.0224749)
Supplement: S5 Appendix — This is the code book describing all the codes from the dataset. (DOCX) [file pone.0224749.s005.docx]

Code Book – Male Clinics, II

| Clinic Location (clinic)  1=Lihanda  2=Marenyo  3=Nyawara  4=Ramula  5=Bar Sauri  6=Yala  7=Mindhine  8=Masogo  9=Onding  10=Gongo  .=Missing | | Sequence Number (SEQN) | |  |
| --- | --- | --- | --- | --- |
| Age at exam (AGE)  .=Missing | | HIV Status Known (STATKNOW)  1=Yes  2=No  .=Missing | |  |
| Reason of Visit (REAS)  1=Checkup  2=Sick  .=Missing | | Services Offered (SERVOFF1)  1=General Physical Assessment  2=Blood Pressure Check  3=STI Screening  4=Other  .=Missing | |  |
| Services Offered (SERVOFF2)  1=General Physical Assessment  2=Blood Pressure Check  3=STI Screening  4=Other  .=Missing | Tests Performed (TEST1)  1=PITC  2=mRDT  3=Bs for MPS  4=VDRL  5=Blood Grouping  6=Hb [Hemoglobin]  7= urinalysis  8= stool examination  9= RBS (random blood sugar)  10=H. Pylori  98=none  99=Other  .=Missing | | |  |
| Tests Performed (TEST2)  1=PITC  2=mRDT  3=Bs for MPS  4=VDRL  5=Blood Grouping  6=Hb [Hemoglobin]  7= urinalysis  8= stool examination  9= RBS (random blood sugar)  10=H. Pylori  98=none  99=Other  .=Missing | Tests Performed (TEST3)  1=PITC  2=mRDT  3=Bs for MPS  4=VDRL  5=Blood Grouping  6=Hb [Hemoglobin]  7= urinalysis  8= stool examination  9= RBS (random blood sugar)  10=H. Pylori  98=none  99=Other  .=Missing | | |  |
| Artemether/Lumefantrine (anti-malarial)_________ (AL)  1=Yes  2=No | | Cotrimoxazole_________ (CTX)  1=Yes  2=No  _ | |  |
| Tetracycline eye ointment_________ (TEO)  1=Yes  2=No | | Doxycycline (DOXY)  1=Yes  2=No | |  |
| Albendazole_________ (ABZ)  1=Yes  2=No | | | Paracetamol/Ibuprofen (PCM)  1 = Yes  2 = No | |
| Erythromycin (ERT)  1=Yes  2=No | | | Multivitamin________ (MVIT)  1=Yes  2=No | |
| Diclofenac________ (DICLO)  1=Yes  2=No | | | Ampiclox (ACLOX)  1=Yes  2=No | |
| Amoxicillin (AMXL)  1=Yes  2=No | | | Metronidazole (MTR)  1=Yes  2=No | |
| Other (Other)  1=Yes  2=No | | | Hydrochlorothiozide (HCTZ)  1 = Yes  2 = No | |
| Steroids (SRO)  1 = Yes  2 = No | | | Anti-epileptics (AEP)  1 = Yes  2 = No | |
| Metformin (METF)  1 = Yes  2 = No | | | Piroxicam (PRX)  1 = Yes  2 = No | |
| Floxapen (FLOX)  1 = Yes  2 = No | | | Ciprofloxacin (CIPRO)  1 = Yes  2 = No | |
| Tetanus Toxoid (TT)  1 = Yes  2 = No | | | Ventolin (VENT)  1 = Yes  2 = No | |
| Piriton or Cetrizine (PRTN)  1 = Yes  2 = No | | | Omeprazole (OMEP)  1 = Yes  2 = No | |
| Nifedipine (NIFED)  1=Yes  2=No | | | Anti-fungals (FUNG)  1 = Yes  2 = No | |
| No treatment given (NONE)  1 = Yes  2 = No | | | 1^st^ Visit (VISIT)  1=Yes  2=No  .=Missing | |
| Diagnosis (DIAG1)  1=Malaria only  2=Malaria + other  3=URTI/RTI,  4=STI,  5=Enteric fever  6=Neuritis  7=Gastroenteritis  8=Fungal infections (group together)  9= Hypertension  10= diabetes  11= epilepsy  12= arthritis  13= allergy  14=Injury/Trauma  15=Sexual dysfunction  16=Other  17=UTI  18=PUD  19=Pneumonia, asthma, pleurisy  .=Missing | | | Referred to lab (REFER)  1=Yes  2=No  .=Missing | |

*(Below are the treatments grouped by purpose)*

***INFECTIOUS:***

**Analgesic (Pain Relief):**

Diclofenac

Ibuprofen (IBU)

Piroxicam

Paracetamol (PCM)

**Antibiotics:**

Amoxicillin (AMXL)

Ampiclox (ACLOX)

Ciprofloxacin (CIPRO)

Doxycycline (DOXY)

Erythromycin (ERT)

Floxapen (FLOX)

Metronidazole (MTR)

Tetracycline eye ointment (applied to newborns to prevent gonorrhea transmission) also conjunctivitis/pink eye)

**Antipyretic (fever reducer):**

Ibuprofen (IBU)

Paracetamol (PCM)

**Dewormer:**

Albendazole (ABZ)

**Vaccine:**

Tetanus

***CHRONIC CONDITIONS:***

**Allergies/antihistamines:**

Cetrizine (CRZN)

Piriton (PRTN)

**Anti-epileptics (neuro)**

**Diabetes:**

Metformin (METF)

**Hypertension:**

Hydrochlorothiozide (HCTZ)

**Respiratory (asthma, COPD):**

Ventolin (VENT)

Steroids (inflammation/respiratory)
